# Effect of smoking on the association of *HHEX* (rs5015480) with diabetes among Korean women and heavy smoking men

**DOI:** 10.1186/s12881-018-0582-0

**Published:** 2018-05-02

**Authors:** Jae Woong Sull, Tae Yong Lee, Sun Ha Jee

**Affiliations:** 1 Department of Biomedical Laboratory Science, College of Health Sciences, Eulji University, Seongnam, South Korea; 2 Department of Preventive Medicine and Public Health, College of Medicine, Chungnam National University, Daejeon, Republic of Korea; 3 Department of Epidemiology and Health Promotion, Institute for Health Promotion, Graduate School of Public Health, Yonsei University, Seoul, South Korea

**Keywords:** Fasting glucose, *HHEX*, Polymorphisms

## Abstract

**Background:**

Several genome-wide association studies (GWAS) for serum fasting glucose levels have reported HHEX as possibly causal. The objective of this study was to examine the joint effect of smoking on the association of diabetes with the *HHEX* rs5015480 polymorphism among Korean subjects.

**Methods:**

This replication study included a total of 4240 individuals, and multivariate linear regression and multiple logistic regression models were used. We examined the combined effect of smoking on the relationship between HHEX rs5015480 and diabetes.

**Results:**

The rs5015480 SNP in the *HHEX* gene was related to the mean FBS level (effect per allele, 1.572 mg/dL, *p* = 0.0122). Females with the CC genotype had a 2.68 times higher (range, 1.05–6.82 times) risk of diabetes than those with the TT/TC genotype. Although the association was stronger in female subjects (OR, 4.46; 95% CI, 1.15–17.3, *p* = 0.0304) among healthy individuals (*N* = 2461), the association between *HHEX* and diabetes was much stronger in male heavy smokers (OR, 4.03; 95% CI, 1.19–13.6, *p* = 0.0247) than in nonsmokers (*p* = 0.9709) and ex-smokers (*p* = 0.2399). The interaction of smoking was also statistically significant (P for interaction =0.0182).

**Conclusions:**

This study clearly demonstrates that a genetic variant in *HHEX* influences fasting glucose levels in Korean women and male heavy smokers.

## Background

The serum fasting glucose level is a basic indicator of diabetes [1], and several genome-wide association studies (GWAS) examining type 2 diabetes have reported that the hematopoietically expressed homeobox (*HHEX*) (MIM 604420) gene is a candidate causal gene [2, 3].

Recent studies have shown that the *HHEX* rs5015480 SNP was related to serum fasting glucose levels or type 2 diabetes [4–9]. In a recent meta-analysis, the rs5015480 SNP was associated with fasting glucose (*p* = 0.015) [4]. Another recent study in the Greek-Cypriot population also reported that the rs5015480 SNP was related to type 2 diabetes (*p* = 0.002) [6]. Several other studies in Chinese population reported that the rs5015480 SNP was related to type 2 diabetes [7–9]. A study in Korean populations reported that the rs5015480 SNP in *HHEX* was related with diabetes [10]. The *HHEX* gene has also been linked with fetal cardiac development [11]. A Chinese study also reported that HHEX rs5015480 was associated with diabetes and cardiovascular risk [12]. However, several other studies reported that rs5015480 was not related to type 2 diabetes [13, 14]. Previous studies did not investigate the relationship between rs5015480 and type 2 diabetes considering smoking status.

Therefore, the objective of this study was to examine the joint effect of smoking on the association of diabetes with the *HHEX* rs5015480 polymorphism among Korean subjects. The relationship between the *HHEX* rs5015480 polymorphism and cardiovascular disease was also examined.

## Methods

### Study population

The participants were 4294 individuals who visited Health Examination Centers from 1994 to 2012 [15]. Among 4294 individuals, 1810 individuals were Cardiovascular Disease (CVD) cases identified by the health insurance reimbursement data from the NHIC. CVD was defined according to the International classification of Disease, Tenth Revision (ICD-10) (I00-I99). In total, 54 individuals were excluded due to missing fasting blood glucose levels and SNP rs5015480 data. The final subjects were 4240 individuals. Among 4240 individuals, 2461 individuals were healthy individuals, and the other 1779 individuals were Cardiovascular Disease (CVD) patients.

### Data collection

The participants were interviewed using a questionnaire about smoking status and smoking amount. Self-reported alcohol consumption data were also collected from the questionnaire. Total cholesterol, high density lipoprotein cholesterol, low density lipoprotein cholesterol, triglyceride, and fasting blood sugar (FBS) were measured from blood samples obtained from each subjects after 12 h of fasting. Height and weight were measured with subjects lightly clothed. Detailed phenotypic data were previously described [15]. Diabetes was defined as fasting serum glucose ≥126 mg/dL or its management under medication.

### Genotyping assays

The rs5015480 HHEX gene SNP was genotyped using the TaqMan reaction [16], with genotyping success rates of more than 98% and repeatability rates of more than 99%.

### Statistical analysis

Data are expressed as means ± standard deviation. Most statistical analyses were performed using PLINK and SAS ver. 9.2 (SAS Institute, Cary, NC, USA). The linear regression under the additive genetic model was used to assess the association of HHEX rs5015480 with fasting blood glucose levels considering age and sex as covariates. We also used multiple logistic regression analysis under the recessive model to examine the combined effect of smoking on the relationship between HHEX rs5015480 and diabetes. Odds ratios (ORs) with 95% confidence intervals (CIs) were calculated to investigate the relationship between *HHEX* SNP and diabetes. In the logistic model, we tested for interactions by assessing the statistical significance of the interaction term in models that included the main effects. A two-sided significance level of α = 0.05 was used.

## Results

The majority of subjects examined were middle-aged individuals (Table 1). Mean FBS level was significantly higher in males (98.9 mg/dL) than in females (93.1 mg/dL) (*p* < 0.0001). About 9.0% of the subjects were diabetes patients, and 14.5% of the subjects had a family history of diabetes. Of the sample dataset, 37.4% of males and 3.8% of females were current smokers, and 44.7% of males and 36.0% of females were cardiovascular disease patients. Table 2 indicates linear regression results, after adjusting for age and sex. The rs5015480 SNP in the *HHEX* gene was related to a mean FBS level (effect per allele, 1.572 mg/dL, *p* = 0.0122).

**Table 1:** General characteristics of the study population

Subjects		All	Men	Women
*N*		4240	2887	1353
		Mean ± SD	Mean ± SD	Mean ± SD
Age, year		52.2 ± 10.2	51.9 ± 10.2	52.7 ± 10.2
Waist circumference, cm		84.1 ± 9.0	87.1 ± 7.6	78.6 ± 8.7
Body mass index, kg/m^2^		24.4 ± 2.9	24.8 ± 2.7	23.5 ± 3.2
Fasting blood sugar, mg/Dl		97.0 ± 22.7	98.9 ± 24.1	93.1 ± 18.7
Systolic blood pressure, mmHg		121.9 ± 14.4	123.3 ± 13.7	118.9 ± 15.3
Diastolic blood pressure, mmHg		78.3 ± 10.8	79.8 ± 10.6	75.0 ± 10.5
HDL cholesterol, mg/Dl		50.9 ± 11.6	48.2 ± 10.2	56.7 ± 12.4
LDL cholesterol, mg/Dl		116.8 ± 31.8	116.6 ± 31.7	117.1 ± 31.9
Triglyceride, mg/Dl		143.8 ± 96.5	157.2 ± 100.9	114.9 ± 78.9
		%	%	%
Smoking status	Ex	28.4	40.1	2.4
	Current	27.0	37.4	3.8
Cardiovascular disease		41.9	44.7	36.1
Diabetes^a^		9.0	10.2	6.3
Family history of diabetes		14.5	14.2	15.3

*SD* standard deviation

^a^Diabetes were defined as fasting serum glucose ≥126 mg/dL or medication

**Table 2:** Association between the rs5015480 single nucleotide polymorphism in the *HHEX* gene and fasting blood sugar levels based on a linear regression model

	Genotypes				
Phenotypes	TT	TC	CC	Effect	*P*-value
	Mean ± SD	Mean ± SD	Mean ± SD	(β)	
All subjects	(*N* = 2827)	(*N* = 1278)	(*N* = 135)		
Fasting blood sugar, mg/dL	96.5 ± 21.7	97.8 ± 24.1	100.1 ± 28.1	1.572	0.0122
Systolic blood pressure, mmHg	121.6 ± 14.0	122.6 ± 15.2	121.6 ± 14.9	0.721	0.0676
Diastolic blood pressure, mmHg	78.3 ± 10.7	78.5 ± 11.1	75.6 ± 10.9	−0.341	0.2541
Healthy subjects	(*N* = 1672)	(*N* = 720)	(*N* = 69)		
Fasting blood sugar, mg/dL	94.0 ± 18.9	95.9 ± 21.8	94.8 ± 19.6	1.420	0.0545
Systolic blood pressure, mmHg	119.2 ± 12.9	119.9 ± 13.8	119.3 ± 12.4	0.544	0.2585
Diastolic blood pressure, mmHg	78.7 ± 10.8	79.0 ± 10.7	76.9 ± 10.0	−0.044	0.9103

Estimated effect size (β) and *p*-value in the multiple linear regression model considered age and sex in the additive model

The relationship of the *HHEX* gene SNP rs5015480 with diabetes was examined (Table 3). Females with the CC genotype had a 2.68 times higher (range, 1.05–6.82-fold) risk of diabetes than those with the TT/TC genotype. For healthy individuals (*N* = 2461), the relationship was stronger in female subjects (OR, 4.46; 95% CI, 1.15–17.3, *p* = 0.0304). In contrast, a relationship between *HHEX* and diabetes was not found in male subjects. The relation of the *HHEX* gene SNP rs5015480 with cardiovascular disease was also examined (Table 4). Females with the CC genotype had a 2.14 times higher (range, 1.05–6.80-fold) risk of cardiovascular disease than those with the TT/TC genotype.

**Table 3:** Odds ratios (OR) of the polymorphic rs5015480 HHEX genotypes for diabetes^a^ in the population

Gene			Normal	Diabetes ^c^		
SNP	Subjects	Genotype	*N* (%)	*N* (%)	OR (95% CI^b^)	*P*-value
HHEX	All	TT/TC	3741 (96.9)	364(95.8)	1.00 (reference)	
rs5015480	(*n* = 4240)	CC	119 (3.1)	16(4.2)	1.44(0.83–2.48)	0.1915
	Men	TT/TC	2509 (96.8)	285(96.6)	1.00 (reference)	
		CC	83 (3.2)	10(3.4)	1.10(0.56–2.17)	0.7736
	Women	TT/TC	1232 (97.2)	79(92.9)	1.00 (reference)	
		CC	36 (2.8)	6(7.1)	2.68(1.05–6.82)	0.0389
	All Healthy		2235 (97.2)	157(97.5)	1.00 (reference)	
	(n = 2461)	CC	65 (2.8)	4(2.5)	0.82(0.29–2.31)	0.7035
	Men	TT/TC	1426 (96.7)	121(99.2)	1.00 (reference)	
		CC	48 (3.3)	1(0.8)	0.24(0.03–1.73)	0.1545
	Women	TT/TC	809 (97.9)	36(92.3)	1.00 (reference)	
		CC	17 (2.1)	3(7.7)	4.46(1.15–17.3)	0.0304

^a^Adjusted for age and sex

^b^CI, confidence interval

^c^Diabetes were defined as fasting serum glucose ≥126 mg/dL or medication

**Table 4:** Odds ratios (OR) of the polymorphic rs5015480 HHEX genotypes for cardiovascular disease^a^ in the population (*n* = 4240)

Subjects		Normal	Cardiovascular Disease		
	Genotype	*N* (%)	*N* (%)	OR (95% CI^b^)	*P*-value
All	TT/TC	2392 (97.2)	1713 (96.3)	1.00 (reference)	
	CC	69 (2.8)	66 (3.7)	1.41 (0.99–2.00)	0.0607
Men	TT/TC	1547 (96.9)	1247 (96.6)	1.00 (reference)	
	CC	49 (3.1)	44 (3.4)	1.17 (0.76–1.79)	0.4735
Women	TT/TC	845 (97.7)	466 (95.5)	1.00 (reference)	
	CC	20 (2.3)	22 (4.5)	2.14 (1.12–4.10)	0.0211

^a^Adjusted for age and sex

^b^CI, confidence interval

The analysis according to smoking status in males is presented in Table 5. The relationship between *HHEX* and diabetes was much stronger in male heavy smokers (OR, 4.03; 95% CI, 1.19–13.6, *p* = 0.0247) than in non-smokers (*p* = 0.9709) and ex-smokers (*p* = 0.2399). Table 6 presents the age-adjusted odds ratios (ORs) for diabetes according to *HHEX* (rs5015480) genotype in strata of smoking status among Korean men. When compared with non-, ex-, or light smokers with the TT/TC genotype, the ORs (95% confidence interval (CI)) were 4.95 (1.51–16.3) in heavy smokers having the CC genotype (P for interaction =0.0182).

**Table 5:** Odds ratios (OR) of polymorphic rs5015480 HHEX genotypes for diabetes^a^ in Korean men (*n* = 2887)

		Normal	Diabetes^c^		
Subjects	Genotype	*N* (%)	*N* (%)	OR (95% CI^b^)	*P*-value
Non smokers	TT/TC	533(97.3)	68(97.1)	1.00 (reference)	
	CC	15(2.7)	2(2.9)	1.03(0.23–4.67)	0.9709
Ex smokers	TT/TC	958(97.2)	113(99.1)	1.00 (reference)	
	CC	28(2.8)	1(0.9)	0.30(0.04–2.24)	0.2399
Light smokers	TT/TC	409(94.0)	36(94.7)	1.00 (reference)	
(1–19/day)	CC	26(6.0)	2(5.3)	0.83(0.19–3.69)	0.8040
Heavy smokers	TT/TC	452(97.8)	50(92.6)	1.00 (reference)	
(≥20/day)	CC	10(2.2)	4(7.4)	4.03(1.19–13.6)	0.0247

^a^Adjusted for age

^b^CI, confidence interval

^c^Diabetes were defined as fasting serum glucose ≥126 mg/dL or medication

**Table 6:** Age-adjusted odds ratios (OR) for diabetes^a^ according to HHEX (rs5015480) genotypes in strata of smoking status in Korean men (*n* = 2887)

	No. of subjects by genotypes	OR (95% CI^b^)		
Subjects		TT/TC	CC	*P* for interaction
Smoking status				0.0182
Non/Ex/Light smokers	2117/74	1.00 (reference)	0.65 (0.26–1.64)	
Heavy smokers	502/14	1.21 (0.87–1.69)	4.95 (1.51–16.3)	

^a^Adjusted for age

^b^CI, confidence interval

## Discussion

In this study of 4240 individuals, the rs5015480 polymorphism in the *HHEX* was related to serum glucose level, which is similar to the results of previous studies. Our study found that *HHEX* polymorphism had a stronger relation to fasting glucose levels in women than in men. However, the interaction between sex and genotypic covariates was not significant (p for interaction = 0.1359) (Data not shown). A study in Korea also reported that the HHEX rs5015480 polymorphism was associated with the risk of diabetes in women (*p* < 0.005), but not in men (*p* > 0.005) [10]. Another recent study suggested the association of the HHEX gene rs5015480 polymorphism with risk of gestational diabetes mellitus in women [17]. In this study, females with the CC genotype had a 2.14 times higher (range, 1.05–6.80-fold) risk of cardiovascular disease than those with the TT/TC genotype. However, an association between *HHEX* and cardiovascular disease was not found in male subjects.

Smoking is also highly related to type 2 diabetes [18–20]. In the present study, 37.4% of males and 3.8% of females were current smokers, which is similar to the results of Korean national data (44.6% male and 4.6% female current smokers) [21]. We also found the association between the *HHEX* SNP and fasting glucose was stronger in heavy smokers than in nonsmokers. Some studies have reported that fasting glucose is regulated by smoking [22, 23]. A recent study reported that genetic polymorphism in glucagon may be modified by smoking for the risk of type 2 diabetes [22]. Another recent study reported that the AMPKα1 polymorphism may have the joint effects with cigarette smoking for the risk of coronary artery disease in the Chinese people [23]. A recent study also suggested interactions of well-known obesity-related polymorphisms with smoking [24].

*HHEX* encode homeobox transcription factors that are involved in organogenesis of liver and pancreas [17, 25]. A recent study reported that HHEX was related to insulin processing and secretion [4]. The frequency of rs5015480 C allele is 56.8% in Europeans and 56.7% in sub-Saharan Africans while the frequency in East Asians was 21.1% in Han Chinese in Beijing and 20.0% in Japanese, as shown in the HapMap data (NCBI website). Our study found a C allele frequency of 18.2%.

In this study, the rs5015480 SNP was related to a mean FBS level (*p* = 0.0122). When the population is restricted to healthy subjects, the significant association was not found (*p* = 0.0545). One of the possible reasons for the difference is that the mean FBS level was lower in the healthy subjects (94.6 mg/dL) than in all subjects (97.0 mg/dL). This study has several other limitations. The available data do not allow us to classify participants by diabetes type. However, the proportion of type I diabetes in Korea is low, at 1% of diabetes cases [26]. In addition, we did not consider other major genes that contribute to type 2 diabetes susceptibility.

## Conclusion

Genetic backgrounds in Western populations were different for Asian populations [27]. Thus, results of this study may not be generalized to all populations. However, our study showed that the *HHEX* gene on chromosome 10 is related to serum glucose levels in Korean women and male heavy smokers.

## Abbreviations

CI: Confidence intervals
CVD: Cardiovascular Disease
FBS: Fasting blood sugar
GWAS: Genome-wide association studies
HDL: High density lipoprotein
HHEX: Hematopoietically expressed homeobox
ICD: International classification of Disease
LDL: Low density lipoprotein
OR: Odds ratios
SD: Standard deviation
SNP: Single nucleotide polymorphism
